# Immediate Induction Versus Expectant Management in Term Premature Rupture of Membranes: A Longitudinal Analysis of Maternal and Neonatal Outcomes

**DOI:** 10.7759/cureus.87640

**Published:** 2025-07-09

**Authors:** Basma Alansari, Hessa Almiraisi, Rehab Alani, Yasmin Abozenah, Maha Ghorabah

**Affiliations:** 1 Obstetrics and Gynecology, King Hamad University Hospital, Busaiteen, BHR; 2 Medicine, Yale School of Medicine, New Haven, USA

**Keywords:** expectant management, immediate induction, induction of labour, outcomes, premature rupture of membranes

## Abstract

Background and aim

Premature rupture of membrane (PROM) is an obstetric problem that refers to the rupture of the membrane integrity of the fetus before delivery. PROM has considerable adverse impacts on both maternal and neonatal outcomes. This study aimed to compare the effectiveness of the two major management strategies for PROM, expectant management and labor induction, by evaluating outcomes between the two groups. This study was conducted due to the lack of previous research comparing these approaches in the context of PROM in Bahrain.

Methods

A prospective longitudinal, non-randomized, comparative interventional study was conducted at the Obstetrics and Gynecology Department of King Hamad University Hospital from July 2021 to December 2022 on pregnant women with a gestational age of 37 weeks or more with confirmed membrane rupture. Demographics, medical, surgical, and obstetric histories were recorded. The study included two baseline-matched groups; Group I involved 86 women who underwent expectant management for 24 hours followed by oxytocin augmentation, whereas Group II included 58 women who underwent immediate induction of labour with prostaglandin.

Results

There were significant differences between the two groups regarding mean age (p = 0.034), parity (p = 0.002), admission to the neonatal intensive care unit (p = 0.025), mode of delivery (p = 0.040), Bishop score (p = 0.003), and induction interval to active labour and delivery (p < 0.0001).

Conclusion

The comparison between immediate induction with prostaglandin and expectant management followed by oxytocin augmentation revealed that there was no definite optimum management approach, as each method has its own benefits and complications. The determination of the best approach can be based on the condition of pregnant women.

## Introduction

Spontaneous rupture of membrane (SROM) occurs in 90% as a normal physiological event, whereas almost 10% of the population experiences it before labour begins [1]. Pre-labour or premature rupture of membrane (PROM) is an obstetric problem with an obscure etiology [2]. PROM is defined as the rupture of the integrity of the membrane of the fetus before the onset of delivery, resulting in amniotic fluid leakage [3,4]. The risk factors of PROM include uterine overdistention, trauma, history of prior PROM, incompetent cervix, infection, preterm labour, previous cervical surgery, amniocentesis, poor nutrition, and connective tissue diseases [5]. PROM can affect both term and preterm pregnancies [4]. It can be classified into three groups based on the gestational age; term PROM occurs after 37 weeks of gestation, whereas preterm PROM and mid-trimester PROM occur between 28-37 and 16-27 weeks of gestation, respectively [2]. PROM occurs among almost 10% of all births, and in 7% and 3% of term and preterm pregnancies, respectively [6]. PROM has a potential impact on maternal and neonatal outcomes, and it complicates the pregnancy with considerable morbidity [7]. The maternal complications that are most commonly associated with PROM include acute and subclinical chorioamnionitis, postpartum endometritis, and placental separation, whereas fetal and neonatal complications include non-reassuring fetal heart rate, hyaline membrane disease, cerebral palsy, pulmonary hypoplasia, fetal deformities, and congenital abnormalities [2]. It is believed that PROM elevates the risk of infection, and hence, induction of delivery is recommended as an attempt to reduce the risk [8]. There are two major approaches to managing PROM, and they are either waiting for the spontaneous onset of labour, and this approach is known as expectant management, or inducing labour with prostaglandins or oxytocin, and this intervention is known as active induction [9]. However, there is controversy about whether the induction decreases the risk of infection [8]. Thus, since the 1960s, there has been debate over how to treat pre-labour rupture of membranes. The debate was centered on whether to induce labour as soon as possible, thus lowering the risk of infection, or to wait for natural initiation of labour to increase the likelihood of natural birth and lower the risk of cesarean section [10]. Although previous studies have been conducted on the current subject, to the best of our knowledge, there was no study in Bahrain comparing immediate induction with expectant management in patients with PROMs; therefore, this study was undertaken.

## Materials and methods

Study design, setting, and subjects

This is a prospective longitudinal, non-randomized, comparative interventional study that was conducted at the Obstetrics and Gynecology Department of King Hamad University Hospital (KHUH) from July 2021 to December 2022. It involved pregnant women with a gestational age of 37 weeks or more without any external complications, those with confirmed membrane rupture, reassuring cardiotocography, cephalic presentation, and a Bishop score of less than 6. The exclusion criteria included any pregnant woman who underwent induction of labour at less than 37 weeks, required direct induction of labour in view of other reasons (fever, or meconium-stained liquor), had a previous cesarean section scar, multiple gestation or had any indications for cesarean section such as placenta previa or breech presentation. The collected data included demographics, medical, surgical, and obstetric histories, physical examination, duration and method of induction, and maternal and neonatal outcomes.

Procedure

Study participants were selected based on the inclusion criteria. The participants were divided into two groups: Group I participants who had expectant management followed by delayed induction of labour after 24 hours by oxytocin augmentation if they did not progress into labour spontaneously. Whereas group 2 participants involved those who had immediate induction in labour by prostaglandin after confirmed Group B streptococcus (GBS) negative status. One cycle of prostaglandin (two doses) was provided to each woman in Group II for labour induction, and reassessment was done after 24 hours if the patient did not progress in labour. If induction with prostaglandin cycle was unsuccessful, pregnant women were shifted to the labour room and were started on oxytocin augmentation according to the department protocol. In case of safety and well-being concerns at any point of the research, whether to the mother or the fetus, the study was abandoned, and the patient was treated according to the departmental protocol.

Statistical analysis

IBM SPSS Statistics for Windows, Version 25 (Released 2017; IBM Corp., Armonk, New York, United States) was used for data analysis; mean (standard deviation) was used to represent numerical variables, whereas number (%) was used to represent categorical variables. The t-test or chi-square test was used for comparison of variables based on the type of data.

## Results

A total of 144 women were included, 86 were involved in Group I as an expectant management group for 24 hours, followed by oxytocin augmentation, and 58 women were enrolled in Group II, who received prostaglandin and were assigned to the immediate induction group. Both groups using t-test were matched in baseline characteristics, where the demographics of women, including weight, BMI, and gestational age, displayed no significant variation between the two groups. On the other hand, only age was significant (p = 0.034) (Table 1).

**Table 1:** Comparison between study groups regarding mother quantitative variables

	Group I		Group II		T-test	P-value
	Mean	Standard deviation	Mean	Standard deviation		
Age	29	4.8	27.2	5.1	2.136	0.034
Weight	72	17.4	72.1	15.6	-0.041	0.967
BMI	28.8	6.1	29	5.4	-0.154	0.878
Gestational age	38.8	1.1	38.7	1	0.127	0.899

The comparison between the two groups using the chi-square test exhibited no significant variation regarding pre-induction cardiotocography and GBS infection. However, parity was significant (p = 0.002) (Table 2).

**Table 2:** Comparison between study groups regarding mother qualitative variables GBS: Group B streptococcus

		Group I		Group II		χ² value	P-value
		Count	%	Count	%		
Pre-induction cardiotocography	Reactive	86	100	58	100	-	-
GBS infection	Negative	86	100	58	100	-	-
	Positive	0	0	0	0	-	-
Parity	Primi	41	47.70	44	75.90	-	-
	Para 1 to 3	37	43.00	13	22.40	12.083	0.002
	More than para 3	8	9.30	1	1.70	-	-

The comparison between the two groups regarding the qualitative variables of delivery and neonates is shown in Table 3. Using the chi-square test, there were no significant variations between both groups regarding the need for tocolysis, uterine hyperstimulation, and postpartum hemorrhage. On the other hand, a significant number of neonates in Group I required admission to the neonatal intensive care unit (NICU) (p = 0.025). In addition to that, a significant proportion of Group I underwent spontaneous vaginal delivery compared to Group II (p = 0.040).

**Table 3:** Comparison between study groups regarding delivery and neonatal qualitative variables NICU: neonatal intensive care unit; CS: caesarean section; SVD: spontaneous vaginal delivery

		Group I		Group II		χ² value	P-value
		N	%	N	%		
Need for tocolysis	No	84	97.70	57	98.30	0.061	0.804
	Yes	2	2.30	1	1.70		
Uterine hyperstimulation	No	84	97.70	57	98.30	0.061	0.804
	Yes	2	2.30	1	1.70		
Post-partum hemorrhage	No	85	98.80	57	98.30	0.08	0.778
	Yes	1	1.20	1	1.70		
Post-partum pyrexia	No	86	100	58	100	-	-
	Yes	0	0	0	0		
NICU admission	No	73	84.90	56	96.60	5.054	0.025
	Yes	13	15.10	2	3.40		
Mode of delivery	CS	13	15.10	19	32.80	6.438	0.04
	SVD	69	80.20	36	62.10		
	Vacuum delivery	4	4.70	3	5.20		

The comparison between the two groups regarding quantitative variables is shown in Table 4. There were no significant differences between the two groups regarding birth weight and duration of hospital stay. Group I tended to have a significantly higher mean Bishop score (p = 0.002). Induction to active labour was significantly longer for Group II compared to Group I (p < 0.0001). Also, induction to delivery was significantly higher for Group II compared to Group I (p < 0.0001).

**Table 4:** Comparison between study groups regarding delivery and neonatal quantitative variables

		Group I	Group II	T-test value	P-value
Bishop score (at 12 hours)	Mean	1.5	1.2	3.108	0.002
	Standard deviation	0.6	0.6		
Induction to active labour (hours)	Mean	5	11	-6.627	p < 0.0001
	Standard deviation	3.5	7.1		
Induction to delivery (hours)	Mean	8.3	15.3	-6.331	p < 0.0001
	Standard deviation	4.9	8.2		
Birth weight	Mean	3.1	3	0.646	0.52
	Standard deviation	0.3	0.4		
Hospital stay (duration in days)	Mean	2.2	2.4	-1.023	0.308
	Standard deviation	0.8	0.6		

## Discussion

PROMs at term cause spontaneous labor in approximately 60% of cases within 24 hours. However, the risk of perinatal morbidity rises with the length of time after rupture of membranes. The question remains whether early induction would provide an extra benefit [11]. Therefore, we conducted this study to compare immediate induction and expectant management followed by oxytocin augmentation of PROM, as there was no study in Bahrain reported on this subject before, to the best of our knowledge.

Upon reviewing the literature, a previous study reported similar findings to our present study regarding the gestational age of participants, as there were no significant differences between induced and expectant groups. However, other baseline characteristics of participants, including mean age and parity, were significant in our study between the two groups. Additionally, there was no considerable variation between the groups regarding postpartum hemorrhage, which was similar to our findings. However, in contrast to our findings, there was no significant difference in the mode of delivery between the two groups, and the expectant management group significantly tended to experience longer PROM to delivery intervals [12].

Another study compared immediate induction and expectant management for PROM among nulliparous women and revealed that there was a significant difference between both groups regarding mode of delivery. A significantly higher proportion of those in the expectant management group delivered vaginally. Furthermore, neonatal and maternal complications of the expectant management group were lower compared to those who underwent immediate induction, including neonatal and maternal infections, postpartum hemorrhage, and endometritis [2]. Such findings were comparable to ours, as a significantly higher proportion of the expectant management group experienced spontaneous vaginal delivery; however, in contrast to the previous study, our current study displayed no significant difference regarding postpartum hemorrhage or GBS infection.

The Gupta et al. study also compared the two management options of PROM. In contrast to our findings, there was no significant difference between the two groups concerning the mean Bishop score. Despite the agreement with our findings in terms of the significant variations in the mean duration of membrane rupture to active labour and PROM to delivery time, our immediate group participants experienced considerably longer mean durations, whereas, in the previous study, women in the expectant group did not. However, the current study and the study by Gupta et al. revealed that women in the expectant management group significantly tended to deliver vaginally compared to the immediate group. Also, in agreement with our study, there was no significant difference in the mean birth weight of neonates born to mothers in both groups [13].

Immediate induction is associated with a reduced incidence of sepsis; however, it is also associated with increased rates of cesarean section because of either failure of induction or uterine hyperstimulation and fetal distress [12]. Therefore, pregnant women should be informed of such information, and their opinions should be considered in the management.

In a previous study, it was reported that expectant management is superior over immediate induction in term PROM cases as expectant management followed by delayed induction using oxytocin allowed a good number of females to experience vaginal labour and lowered the number of infectious morbidity of mothers and neonates [2]. Similarly, we found that expectant management was significantly associated with vaginal labour with no significant increase in GBS infection.

In a prospective randomized controlled study that compared active management of PROM at term to expectant management, it was discovered that the two approaches yielded comparable results regarding complications and method of delivery. However, active induction significantly reduced the latency period and induction to delivery interval compared to expectant management [9]. Controversial findings were recorded in the current study as immediate induction was associated with longer intervals of active labour induction and delivery.

Based on a Cochrane systematic review, it was found that maternal infectious morbidity, including chorioamnionitis and/or endometritis, was high in women who underwent expectant management following term PROM, and their neonates were more prone to experience definite or probable early-onset neonatal sepsis combined. However, there was no significant variation regarding cesarean section, maternal mortality and morbidity, and definite neonatal sepsis alone [14]. In our study, we did not assess neonatal sepsis, chorioamnionitis, or endometritis, and both groups had no recorded GBS infection.

## Conclusions

The comparison between immediate induction and expectant management for PROM revealed that expectant management was associated with higher vaginal delivery rates and shorter duration intervals for delivery. However, immediate induction was associated with a lower rate of admission to the NICU. Hence, the optimum management approach cannot be determined as each approach has its own benefits and complications. Therefore, discussing treatment strategies with pregnant women is necessary to earn their satisfaction.
